# Value of adjuvant chemotherapy for patients with pT2N0M0 non‐small cell lung cancer receiving radical resection

**DOI:** 10.1111/1759-7714.15192

**Published:** 2023-12-14

**Authors:** Shiqi Chen, Siqian Yang, Yue Zhao, Yang Zhang, Qingyuan Huang, Haoxuan Wu, Hong Hu, Yihua Sun, Yawei Zhang, Jiaqing Xiang, Ting Ye, Haiquan Chen

**Affiliations:** ^1^ Departments of Thoracic Surgery and State Key Laboratory of Genetic Engineering Fudan University Shanghai Cancer Center Shanghai China; ^2^ Institute of Thoracic Oncology Fudan University Shanghai China; ^3^ Department of Oncology, Shanghai Medical College Fudan University Shanghai China; ^4^ School of Life Sciences Fudan University Shanghai China

**Keywords:** adjuvant chemotherapy, non‐small cell lung cancer, pT2N0M0, radical resection

## Abstract

**Background:**

Associations between adjuvant chemotherapy (ACT) and the improvement in survival for patients with pT2N0M0 non‐small cell lung cancer (NSCLC) who received R0 resection remain controversial. This study aimed to evaluate the value of ACT for patients with pT2N0M0 NSCLCs, and to identify the subgroups who could benefit from ACT.

**Methods:**

Multivariable Cox proportional hazards regression models were used to estimate independent prognostic factors. High‐risk factor (HRF) included visceral pleural invasion (VPI), lymphovascular invasion (LVI) and poor differentiation/undifferentiated tumors.

**Results:**

Of the 899 patients, 277 (30.8%) patients received ACT. More younger patients (*p* < 0.001) and male patients (*p* = 0.007) received ACT. With the increase of pathological tumor size (*p* < 0.001) and the number of HRFs (*p* < 0.001), there was a significant rise in the proportion of patients receiving ACT. For all patients, ACT could not improve recurrence‐free survival (RFS) (*p* = 0.672) and overall survival (OS) (*p* = 0.306). For patients with pathological stage IIA or radiological pure‐solid tumors, ACT could significantly improve the OS (*p* = 0.011 and *p* = 0.037, respectively), and multivariate analysis revealed that ACT was an independent prognostic factor for patients with pathological stage IIA (*p* = 0.005). ACT could improve the OS significantly in patients with pathological stage IB pure‐solid lung adenocarcinoma (LUAD) (*p* = 0.043).

**Conclusion:**

ACT was valuable for patients with pathological stage IIA (pT2bN0M0) and patients with radiological pure‐solid LUAD of pathological stage IB. A combination of radiological features and pathological subtypes could be helpful when selecting patients with pT2N0M0 NSCLCs for ACT.

## Graphical abstract

Patients with pathological stage IIA (pT2bN0M0) or patients with pathological stage IB (pT2aN0M0) pure‐solid lung adenocarcinoma could be suitable candidates for ACT after radical surgery of NSCLC.

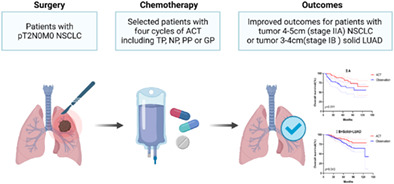

**Abbreviations**
ACT: adjuvant chemotherapy
APA: acinar pattern‐predominant adenocarcinoma
CALGB: Cancer and Leukemia Group B
FUSCC: Fudan University Shanghai Cancer Center
HRF: high‐risk factor
IMA: invasive mucinous adenocarcinoma
LPA: lepidic predominant adenocarcinoma
LUAD: lung adenocarcinoma
LVI: lymphovascular invasion
MPA: micropapillary predominant adenocarcinoma
NCCN: National Comprehensive Cancer Network
NSCLC: non‐small cell lung cancer
OS: overall survival
PPA: papillary pattern‐predominant adenocarcinoma
PSN: part‐solid nodules
RCT: randomized clinical trial
RFS: recurrence‐free survival
SN: solid nodules
SPA: solid predominant adenocarcinoma
SQCC: squamous cell carcinomas
STAS: spread through air spaces
TS‐CT: thin‐section CT scan
VPI: visceral pleural invasion

## INTRODUCTION

The value of adjuvant chemotherapy (ACT) in improving the survival of patients with non‐small cell lung cancer (NSCLC) who received radical surgery has been extensively studied,^1^, ^2^, ^3^, ^4^, ^5^ and the presence of lymph node metastasis has been accepted as an indication for ACT according to the National Comprehensive Cancer Network (NCCN) guidelines.^6^ However, the value of ACT in patients with node‐negative NSCLCs remain controversial, especially in patients with pathologic T2N0M0 diseases.^7^, ^8^, ^9^

In 2008, the multicenter randomized clinical trial (RCT), Cancer and Leukemia Group B (CALGB) 9633, analyzed the value of ACT for patients with stage IB NSCLC diseases and demonstrated that patients with tumors ≥4 cm could benefit from the ACT.^10^ Therefore, tumor size became a common criterion for the use of ACT in patients with node‐negative NSCLCs. In addition, the latest National Comprehensive Cancer Network (NCCN) guidelines defined six high‐risk factors, such as poorly differentiated tumors, visceral pleural invasion (VPI), lymphovascular invasion (LVI), tumors >4 cm, wedge resection, and unknown lymph node status (Nx), for patients with node‐negative stage IB–IIA NSCLCs who might be candidates for ACT.^6^ In 2020, Pathak et al. conducted a retrospective study to explore the associations of survival with ACT among patients with early‐stage NSCLCs stratified by the presence or absence of high‐risk pathological features (VPI, LVI, and poor differentiation or undifferentiated tumors), sublobar surgery, and tumor size.^11^ They found that ACT was not associated with improved survival for patients with tumors ≤3 cm, but associated with a survival benefit among patients with tumors 4–5 cm and only at least one high‐risk pathological feature. Accordingly, patients with these high‐risk factors alone could not benefit from ACT. Furthermore, whether patients with pathological stage IB could benefit from ACT still remain controversial.

Therefore, this study aimed to evaluate the value of ACT for patients with pT2N0M0 NSCLCs who received radical resection and to identify the patient subgroups who might benefit from ACT.

## METHODS

### Patients cohort

This study cohort was retrospectively collected from the department of thoracic surgery at Fudan University Shanghai Cancer Center (FUSCC) from January 2008 to December 2020. Of 17 971 patients with pathologically confirmed lung cancers, 1065 patients with pathological T2N0M0 NSCLCs according to the 8th edition of the WHO TNM classification receiving radical resection were reviewed. The key exclusion criteria were as follows: (1) multiple nodules or prior history of malignant tumors, (2) non‐R0 resection, (3) incomplete follow‐up information, and (4) unknown histological subtypes. The following variables were collected: age, gender, smoking history, radiological features on preoperative thin‐section CT scan (TS‐CT), pathological subtypes, tumor differentiation grade, LVI, VPI, time to last follow‐up, and time to recurrence or death. This research was performed based on the Declaration of Helsinki and approved by the institutional review board of the Fudan University Shanghai Cancer Center. Informed consents were waived because it was a retrospective study.

### Radiological and histological evaluation and definition of High‐risk factors

Radiological solid nodules (SNs) or part‐solid nodules (PSNs) on preoperative TS‐CT of all included patients were identified by two radiologists. LUAD subtypes were classified based on the 2011 IASLC/ATS/ERS LUAD classification. Lepidic predominant adenocarcinoma (LPA) was defined as low‐grade LUAD; acinar predominant adenocarcinoma (APA) / papillary predominant adenocarcinoma (PPA)/invasive mucinous adenocarcinoma (IMA) was defined as intermediate‐grade LUAD; micropapillary predominant adenocarcinoma (MPA) / solid predominant adenocarcinoma (SPA) was defined as high‐grade LUAD. High‐risk factors (HRF) were defined as VPI, LVI, or poor differentiation/undifferentiated tumors. Patients with at least one of the three high‐risk factors were defined as the presence of HRF, and patients with none of the three factors were defined as the absence of HRF.

### Postoperative adjuvant chemotherapy

The platinum‐doublet regimens of ACT were performed individually for patients by oncologists according to NCCN guidelines including TP (taxel + carboplatin/cis‐platinum), NP (vinorelbine + carboplatin/cis‐platinum), PP (pemetrexed + carboplatin/cis‐platinum), and GP (gemcitabine + carboplatin/cis‐platinum). Patients who finished all four cycles of ACT were included in this study.

### Follow‐up protocol

For the first 3 years after surgery, patients underwent regular follow‐ups every 3–4 months. For the next 2 years, follow‐up was performed every 6 months, and once a year in subsequent years. Overall survival (OS) was defined as the time interval between the surgery date to the last follow‐up or death from any cause. Recurrence‐free survival (RFS) was defined as the time interval between the surgery date to the first recurrence or the last follow‐up.

### Statistical analysis

The associations between ACT and clinicopathological and radiological features were assessed using Fisher exact test for categorical variables and Student *t* test for numerical variables. OS and RFS curves were plotted using the Kaplan–Meier method and compared with the log‐rank test. Multivariable Cox proportional hazards regression models were used to estimate the potential independent prognostic factors for OS and RFS. *p*‐values less than 0.05 were considered statistically significant. All statistical analysis was performed using SPSS 25.0 (IBM, Chicago, IL) and GraphPad Prism 8 (GraphPad Software).

## RESULTS

### Patient characteristics

**FIGURE 1:**
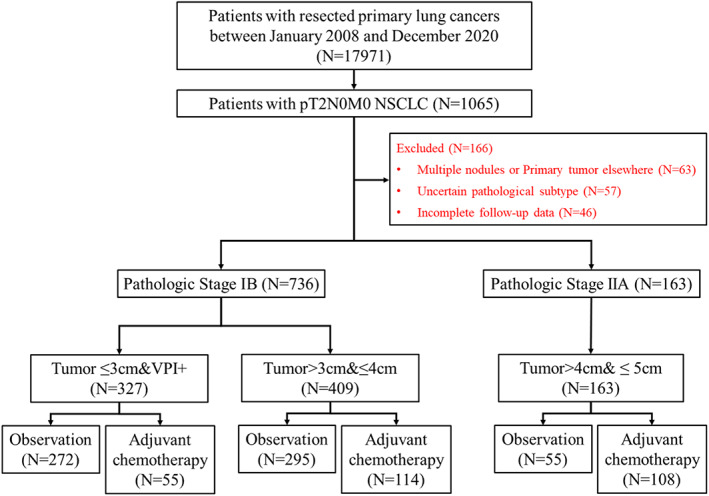
The flow diagram of patient selection in this study.

A total of 899 patients with pathological T2N0M0 NSCLCs who received R0 resection were enrolled in this study. There were 327 (36.4%) patients with tumor ≤3 cm &VPI (+) (pathological stage IB); 409 (45.5%) patients with 3 < tumor size ≤4 cm (pathological stage IB); 163 (18.1%) patients with 4 < tumor size ≤5 cm (pathological stage IIA) (Figure 1).

There were 277 (30.8%) patients who received ACT, and 622 (69.2%) patients who did not receive ACT after surgery. The correlations between ACT and clinicopathological features are listed in Table 1. ACT was performed more in patients of younger age (*p* = 0.001), poor differentiation or undifferentiated tumors (*p* < 0.001), larger sized tumors (*p* < 0.001), the presence of VPI (*p* < 0.001), the presence of LVI (*p* < 0.001), and more HRFs (*p* < 0.001). There were 55 (16.8%), 114 (27.9%), and 108 (66.3%) patients who received ACT in groups of patients with tumor size ≤3 cm and VPI (+), 3 < tumor size ≤4 cm, and 4 < tumor size ≤5 cm, respectively (*p* < 0.001). Radiologically, more patients with SNs received ACT compared with those with PSNs (258 [34.7%] vs. 19 [12.2%], *p* < 0.001).

**TABLE 1:** Clinicopathological characteristics of patients with pT2N0M0 NSCLC (*N* = 899).

Characteristic	Patients, N (%)		*p*‐value
	Observation *N* = 622 (69.2%)	Adjuvant chemotherapy *N* = 277 (30.8%)	
Age (years)			
≤60	216 (62.4%)	130 (37.6%)	0.001
>60	406 (73.4%)	147 (26.6%)	
Gender			
Male	373 (66.0%)	192 (34.0%)	0.007
Female	249 (74.6%)	85 (25.4%)	
Smoking history			
Ever	295 (66.4%)	149 (33.6%)	0.078
Never	327 (71.9%)	128 (28.1%)	
Operative procedure			
Sublobar resection	33 (86.8%)	5 (13.2%)	0.042
Lobectomy	547 (69.3%)	242 (30.7%)	
Sleeve resection	10 (58.8%)	7 (41.2%)	
Bilobectomy	20 (55.6%)	16 (44.4%)	
Pneumonectomy	12 (63.2%)	7 (36.8%)	
p‐TNM stage			
≤3 cm and VPI+	272 (83.2%)	55 (16.8%)	<0.001
IB >3 cm and ≤ 4 cm	295 (72.1%)	114 (27.9%)	
IIA >4 cm and ≤ 5 cm	55 (33.7%)	108 (66.3%)	
Grade			
Well differentiated	23 (100.0%)	0 (0.0%)	<0.001
Moderately differentiated	453 (72.1%)	175 (27.9%)	
Poorly differentiated	146 (58.9%)	102 (41.1%)	
GGO components			
Part‐solid nodules	137 (87.8%)	19 (12.2%)	<0.001
Solid nodules	485 (65.3%)	258 (34.7%)	
Histological type			
LPA	17 (100.0%)	0 (0.0%)	<0.001
APA	253 (75.3%)	83 (24.7%)	
PPA	87 (71.3%)	35 (28.7%)	
IMA	22 (64.7%)	12 (35.3%)	
MPA	10 (52.6%)	9 (47.4%)	
SPA	42 (53.8%)	36 (46.2%)	
SQCC	161 (64.1%)	90 (35.9%)	
Other^a^	30 (71.4%)	12 (28.6%)	
VPI			
Absent	291 (64.1%)	163 (35.9%)	<0.001
>3 cm and ≤5 cm	59 (50.0%)	59 (50.0%)	
Present			
≤ 3 cm	272 (83.2%)	55 (16.8%)	
LVI			
Absent	552 (72.4%)	210 (27.6%)	<0.001
Present	70 (51.1%)	67 (48.9%)	
Number of high‐risk factors^b^			
0	395 (78.5%)	108 (21.5%)	<0.001
1	185 (61.3%)	117 (38.7%)	
2	36 (44.4%)	45 (55.6%)	
3	6 (46.2%)	7 (53.8%)	

Abbreviations: APA, acinar pattern‐predominant adenocarcinoma; GGO, ground‐glass opacity; IMA, invasive mucinous adenocarcinoma; LPA, lepidic predominant adenocarcinoma; LVI, lymphovascular invasion; MPA, micropapillary predominant adenocarcinoma; PPA, papillary pattern‐predominant adenocarcinoma; SPA, solid predominant adenocarcinoma; SQCC, squamous cell carcinoma; VPI, visceral pleural invasion.

^a^ There are 17 patients with large cell carcinoma, 14 patients with adenosquamous carcinoma, nine patients with carcinoid, one patient with mucoepidermoid carcinoma and one patient with fetal pattern–predominant adenocarcinoma.

^b^ High‐risk factors included poorly differentiated or undifferentiated tumors, visceral pleural invasion and lymphovascular invasion.

### Survival analysis

The median follow‐up duration was 59.0 months (range, 55.0–63.0 months). During the follow‐up period, 220 (24.5%) patients experienced postoperative tumor recurrence or metastasis. The 5‐year OS and RFS of all patients were 80.0% (95% CI: 78.0%–82.0%) and 72.0% (95% CI: 70.0%–74.0%), respectively. For patients with stage IB, the 5‐year OS and RFS were 81.0% (95% CI: 79.0%–83.0%) and 72.0% (95% CI: 70.0%–74.0%), respectively. For patients with stage IIA, the 5‐year OS and RFS were 74.0% (95% CI: 70.0%–78.0%) and 72.0% (95% CI: 68.0%–76.0%), respectively.

**FIGURE 2:**
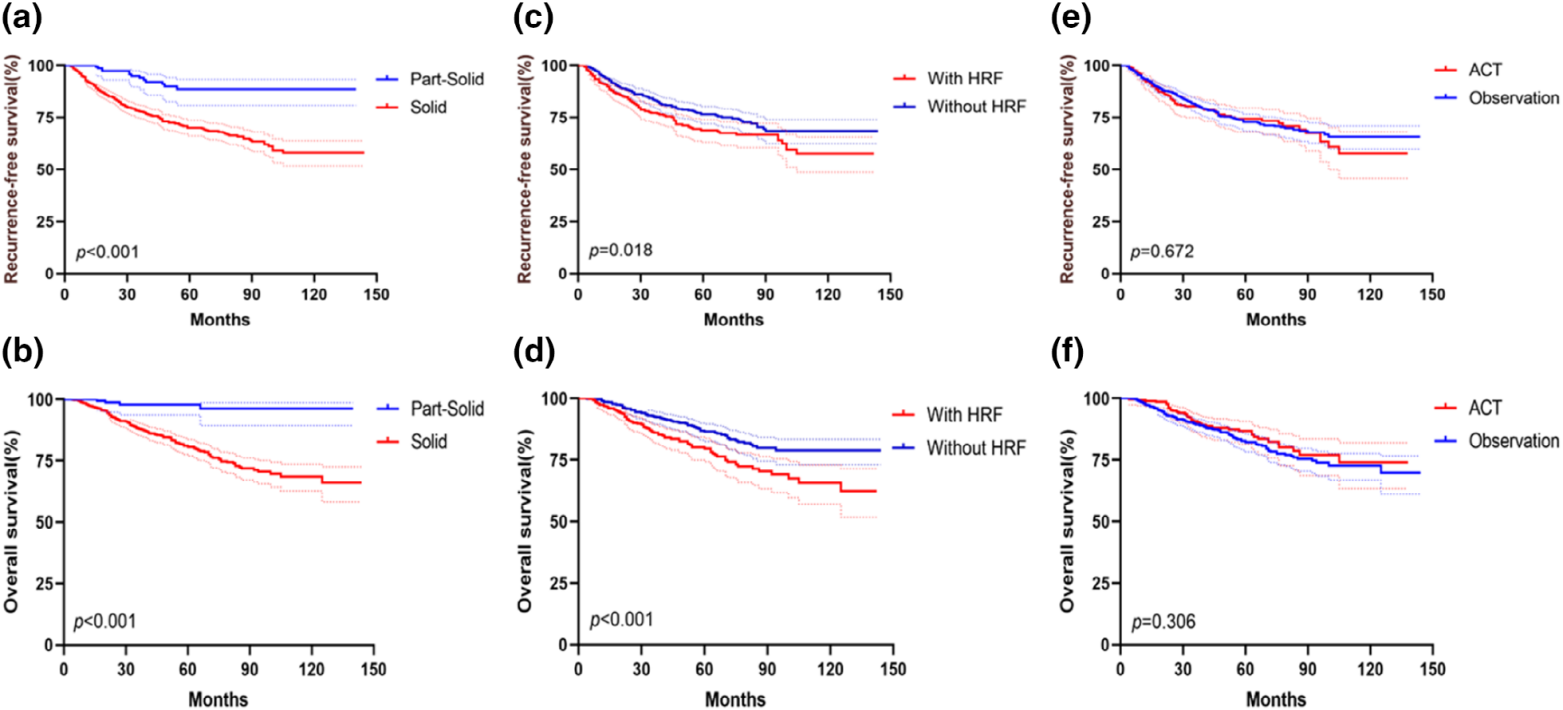
Recurrence‐free survival and overall survival according to radiological feature, the presence of high‐risk factor (HRF) and adjuvant chemotherapy (ACT) in pT2N0M0 non‐small cell lung cancer (NSCLC) patients.

Overall, patients with radiological SNs had a significantly worse RFS (*p* < 0.001) and OS (*p* < 0.001) than those with PSNs did (Figure 2a,b) In addition, patients with the presence of HRF had a significantly worse RFS (*p* = 0.018) and OS than those without any HRFs did (*p* < 0.001) (Figure 2c,d). In multivariable analysis, radiological SNs was an independent prognostic factor for RFS (*p* < 0.001) and OS (*p* < 0.001) (Table 2), but HRF was not an independent prognostic factor for RFS (*p* = 0.289) and OS (*p* = 0.221) (Table 2). Moreover, patients with pT2N0M0 NSCLCs could not benefit from ACT for RFS (*p* = 0.672) and OS (*p* = 0.306) (Figure 2e,f).

**TABLE 2:** Univariate and multivariate analysis of surgical outcomes for patients with pT2N0M0 NSCLCs.

Variables	RFS				OS			
	Univariate analysis		Multivariate analysis		Univariate analysis		Multivariate analysis	
	HR (95% CI)	*p*‐value	HR (95% CI)	*p*‐value	HR (95% CI)	*p*‐value	HR (95% CI)	*p*‐value
Age, years (≥60 vs. <60)	1.387 (1.046–1.839)	**0.023**	1.341 (1.011–1.778)	**0.042**	2.195 (1.500–3.212)	**<0.001**	2.154 (1.471–3.153)	**<0.001**
Gender (male vs. female)	0.885 (0.672–1.166)	0.386			0.659 (0.463–0.939)	**0.021**		0.270
Smoking history (ever vs. never)	1.117 (0.857–1.455)	0.414			1.289 (0.930–1.786)	0.127		
Eighth TNM stage (IIA vs. IB)	1.082 (0.770–1.519)	0.650			1.511 (1.033–2.209)	**0.032**		0.377
GGO component (solid vs. part‐solid)	3.813 (2.177–6.679)	**<0.001**	3.748 (2.140–6.567)	**<0.001**	7.970 (2.951–21.529)	**<0.001**	7.452 (2.757–20.139)	**<0.001**
Histological grade (high vs. low/intermediate)	1.157 (0.864–1.551)	0.328			1.597 (1.134–2.248)	**0.007**		0.354
VPI (positive vs. negative)	1.055 (0.721–1.544)	0.782			1.608 (1.075–2.405)	**0.021**	1.559 (1.041–2.333)	**0.031**
LVI (positive vs. negative)	1.455 (1.027–2.060)	**0.035**		0.066	1.184 (0.744–1.884)	0.475		
High‐risk factors (with vs. without)	1.372 (1.053–1.788)	**0.018**		0.289	1.738 (1.254–2.408)	**<0.001**		0.221
Adjuvant chemotherapy (without vs. with)	0.941 (0.708–1.249)	0.672			1.210 (0.839–1.747)	0.306		

*Note*: Bold indicates more visibility (*p* < 0.05).

Abbreviations: CI, confidence interval; GGO, ground‐glass opacity; HR, hazard ratio; LVI, lymphovascular invasion; NSCLC, non‐small cell lung cancer; OS, overall survival; RFS, recurrence‐free survival; VPI, visceral pleural invasion.

**FIGURE 3:**
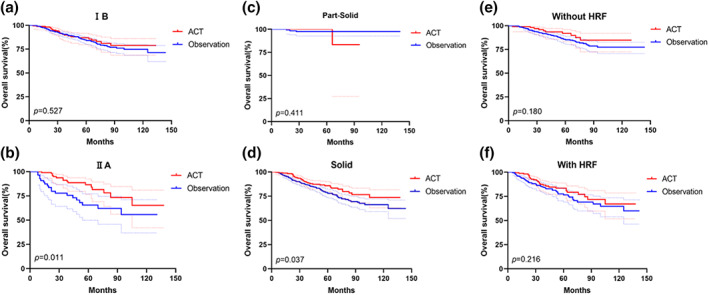
Overall survival according to the absence or presence of adjuvant chemotherapy (ACT) in pT2N0M0 non‐small cell lung cancer (NSCLC) patients stratified by pathological stage, radiological feature and the presence of high‐risk factor (HRF).

Furthermore, we performed the subgroup analysis stratified by pathological stage, radiological feature, and presence of HRFs. ACT could not improve the RFS and OS for patients with stage IB (*p* = 0.526, *p* = 0.527 respectively) (Figure 3a) or PSNs (*p* = 0.652, *p* = 0.411 respectively) (Figure 3c). However, patients with pathological stage IIA (*p* = 0.011) or SNs (*p* = 0.037) could benefit from ACT for OS (Figure 3b,d), but not for RFS (*p* = 0.542, *p* = 0.588 respectively). In multivariate analysis, ACT was an independent prognostic factor for OS in patients with stage IIA (*p* = 0.005). Moreover, ACT could not improve the patients' RFS (with HRF: *p* = 0.754; without HRF: *p* = 0.605) and OS (with HRF: *p* = 0.216; without HRF: *p* = 0.180) (Figure 3e,f) regardless the presence of HRF or not.

**FIGURE 4:**
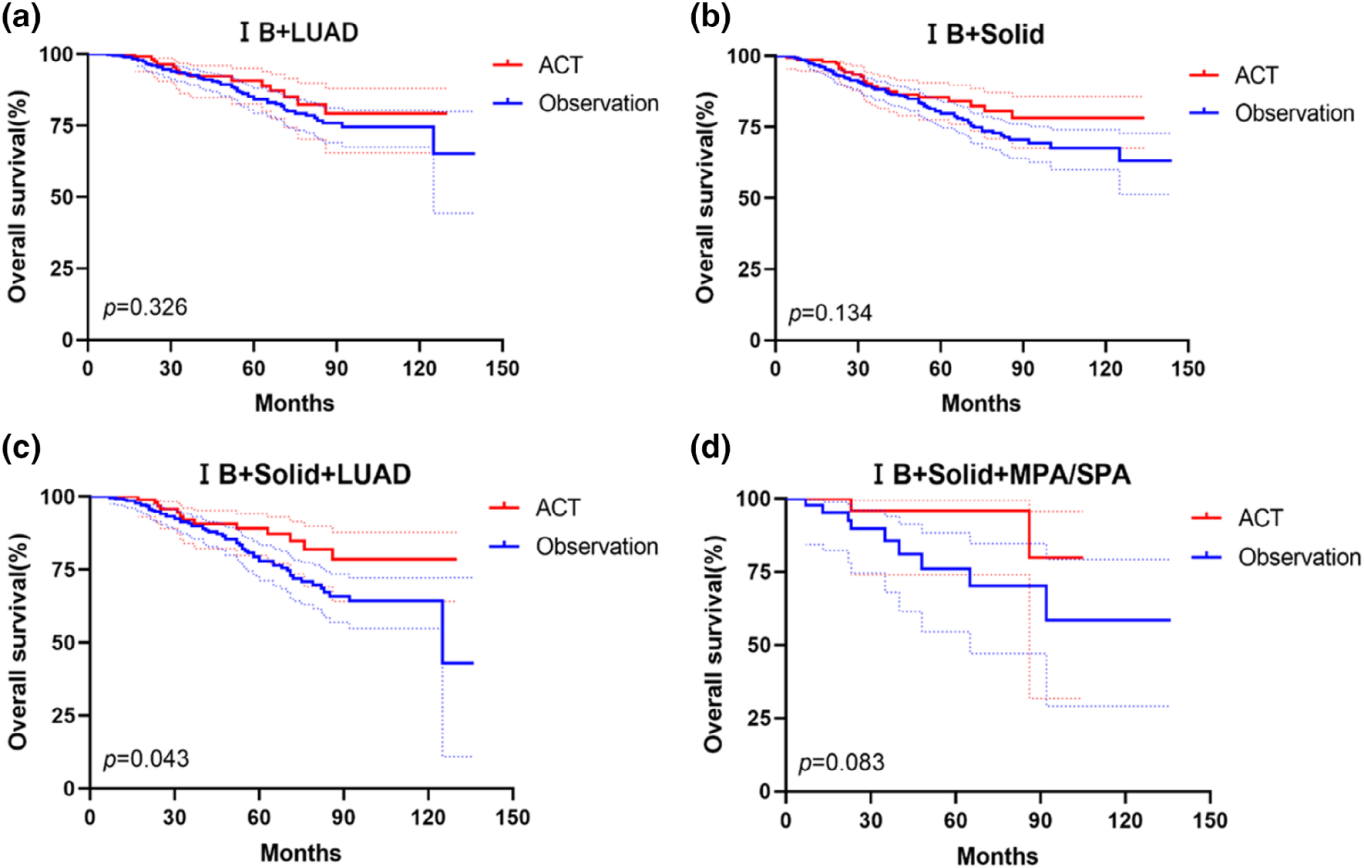
Overall survival according to the absence or presence of ACT stratified by pathological subtypes and radiological feature in stage IB non‐small cell lung cancer (NSCLC) patients.

For patients with pathological stage IB diseases, ACT could not improve the OS in subgroups of patients with LUAD (*p* = 0.326) (Figure 4a) and patients with radiological SNs (*p* = 0.134) (Figure 4b), but ACT could significantly improve the OS for these patients with radiological pure‐solid LUAD (*p* = 0.043) (Figure 4c), for these patients with MPA/SPA (*p* = 0.083) (Figure 4d).

## DISCUSSION

Over the past decade, the value of ACT for patients with pT2N0M0 NSCLCs who receive radical resection, especially for those with pathological stage IB diseases (pT2aN0M0) according to the eighth edition of the WHO TNM classification of NSCLCs, remains controversial. Few RCTs have focused on this issue due to the limited number of enrolled patients and the long follow‐up time. Therefore, it is important to identify the candidates who could benefit from ACT and those who could not benefit from ACT so as to avoid the potentially toxic effects and adverse effects of ACT.

In this study, ACT could not improve the RFS and OS for all included patients. This was consistent with the results of the CALGB 9633 trial. In CALGB 9633 trial, only patients with tumors ≥4 cm could benefit from ACT in subgroup analysis.^10^ Therefore, we conducted subgroup analysis to evaluate the value of ACT in patients stratified by tumor size. Similarly, patients with tumor 4–5 cm (stage IIA) could benefit from ACT, and ACT was an independent prognostic factor for OS in these patients. In 2016, Morgensztern et al. analyzed the role of ACT in a large data set of patients with completely resected T2N0M0 NSCLC (3–7 cm, according to the seventh TNM classification) and reported that ACT was associated with improved survival for patients with T2N0M0 NSCLC in all tumor‐sized groups.^12^ However, in 2020, Pathak et al. reported that a survival benefit associated with ACT for node‐negative tumors 4–5 cm was limited to only patients with at least one high‐risk factor.^11^ Therefore, according to the eighth edition of the WHO TNM classification, tumor size larger than 4 cm has been classified as IIA disease, and tumor size larger than 4 cm could be accepted as an indication for ACT in patients with node‐negative NSCLC.^10^, ^11^, ^12^, ^13^

For patients with pathological stage IB NSCLCs, whether ACT was beneficial remained unconfirmed. In this study, we found that ACT could not improve the RFS and OS for all patients with pathological stage IB diseases. Similarly, Pathak et al. evaluated the association between ACT and survival in the presence and absence of high‐risk pathological features in patients with node‐negative early‐stage NSCLC and reported that ACT was not associated with survival outcomes in patients with tumors between 3 to 4 cm (stage IB) regardless of the presence of HRF or not.^11^ Additionally, in 2020, Park et al. suggested that ACT did not affect the prognosis of stage IB NSCLC, even in high‐risk patients.^14^ Furthermore, in 2019, Wang et al. found ACT could result in worse survival than observation alone for these patients.^15^ Therefore, high‐risk pathological features could not be helpful to select candidates of patients with pathological stage IB for ACT. On the other hand, in 2018, Qian et al. showed that ACT could prolong survival in patients with MPA/SPA of stage IB.^16^ In 2020, another study suggested that tumor spread through air spaces (STAS) could be an indicator of ACT for pathological stage IB patients.^17^ In this study, our results indicated that patients with radiological pure‐solid LUAD could benefit from ACT. Therefore, the clinicopathological features such as pathological subtypes, radiological features and STAS might serve as an important indicator for screening candidates of pathological stage IB for ACT. Accordingly, different results and conclusions from these studies might be resulted from different study periods, different study population size, or different chemotherapeutic agents used. Therefore, one prospective RCT with a large sample size is needed to determine the real value of ACT for patients with pathological stage IB NSCLCs in the future.

Recently, radiomics and pathomics have been applied to identifying high‐risk patients that would be potential candidates for ACT. In 2020, Vaidya et al. developed a 13‐feature‐based quantitative radiomic risk score (QuRiS), which was predictive of the benefit to ACT following radical resection in early‐stage NSCLC.^18^ In 2020, Xie et al. developed an eight‐feature‐consisted radiomics signature to identify the benefit of ACT for patients with resected stage I LUAD.^19^ Wang et al. developed an 11‐feature pathomics predictive signature for ACT benefit in patients with early‐stage NSCLC.^20^ These technologies may potentially facilitate the clinical impact in guiding therapeutic administration. However, this study was not aimed to address this issue.

A limitation of this study was that it was a single‐center retrospective analysis and there was a bias in patient selection for ACT. Because of the lack of ACT guidelines for patients with pT2N0M0 NSCLCs, especially for patients with pathological stage IB diseases, more younger patients and those with postoperatively good conditions who were considered at high‐risk of recurrence were clinically recommended to receive ACT. The strengths of this study were that it had a large sample size and a long follow‐up time compared with previous studies.

In conclusion, ACT was indicated for patients with pathological stage IIA (pT2bN0M0) and patients with radiological pure‐solid stage IB LUADs. A combination of radiological features and pathological subtypes could be valuable for recommending ACT to patients with pT2N0M0 NSCLCs.

## AUTHOR CONTRIBUTIONS

Shiqi Chen and Ting Ye: Conceptualization; Methodology; Data curation; Formal analysis; Writing – original draft; Writing ‐ review & editing. Siqian Yang and Yue Zhao: Methodology; Software; Visualization. Yang Zhang, Qingyuan Huang and Haoxuan Wu: Investigation; Methodology. Hong Hu, Yihua Sun, Yawei Zhang and Jiaqing Xiang: Writing – Review & Editing. Ting Ye and Haiquan Chen: Conceptualization; Supervision; Funding acquisition.

## CONFLICT OF INTEREST STATEMENT

The authors have no relevant financial or nonfinancial interests to disclose.
